# An Integrative Approach to Determine 3D Protein Structures Using Sparse Paramagnetic NMR Data and Physical Modeling

**DOI:** 10.3389/fmolb.2021.676268

**Published:** 2021-08-12

**Authors:** Kari Gaalswyk, Zhihong Liu, Hans J. Vogel, Justin L. MacCallum

**Affiliations:** ^1^ Department of Chemistry, University of Calgary, Calgary, AB, Canada; ^2^ Department of Biological Sciences, University of Calgary, Calgary, AB, Canada

**Keywords:** paramagnetic relaxation enhancement, NMR, modeling, protein structure, integrative structural biology, calmodulin

## Abstract

Paramagnetic nuclear magnetic resonance (NMR) methods have emerged as powerful tools for structure determination of large, sparsely protonated proteins. However traditional applications face several challenges, including a need for large datasets to offset the sparsity of restraints, the difficulty in accounting for the conformational heterogeneity of the spin-label, and noisy experimental data. Here we propose an integrative approach to structure determination combining sparse paramagnetic NMR with physical modelling to infer approximate protein structural ensembles. We use calmodulin in complex with the smooth muscle myosin light chain kinase peptide as a model system. Despite acquiring data from samples labeled only at the backbone amide positions, we are able to produce an ensemble with an average RMSD of ∼2.8 Å from a reference X-ray crystal structure. Our approach requires only backbone chemical shifts and measurements of the paramagnetic relaxation enhancement and residual dipolar couplings that can be obtained from sparsely labeled samples.

## Introduction

Protein nuclear magnetic resonance (NMR) spectroscopy has played an important role in biomolecular structure determination. To date more than 13,000 NMR structures have been deposited in the Protein Data Bank [PDB (Berman et al., 2000)], accounting for about 7.5 percent of all available protein structures (PDB Statistics, 2021). The vast majority of the deposited NMR solution structures are determined for smaller proteins or independently-folded isolated protein domains (Tugarinov et al., 2004). Without special stable isotopic labelling techniques, NMR methods struggle with structure determination of proteins larger than ∼25 kDa as the slow molecular tumbling results in rapid relaxation, leading to poor resolution and spectral quality (Kay, 2016). The most commonly used method to overcome this challenge is to combine transverse relaxation optimized spectroscopy (TROSY) (Pervushin et al., 1997) with site-specific protonation in an otherwise perdeuterated background (Tugarinov et al., 2006), although there are many alternatives, e.g., Tugarinov et al. (2004) and Ruschak and Kay (2010). While such site-specific isotope labelling can dramatically increase the spectral quality and interpretability, the overall perdeuteration results in protons being sparsely distributed within the structure.

The overwhelming majority of solution NMR structures in the PDB are based around Nuclear Overhauser Effect Spectroscopy (NOESY), which provides information about through-space interactions between protons that can be used to derive distance restraints for 3D structure determination. For the homonuclear NOE to be detectable, however, the protons must be within about 6 Å or closer, which can pose a substantial challenge for sparsely-labelled samples due to the lack of proton pairs that are in close proximity within the folded protein structure (Gardner and Kay, 1998).

This phenomenon is particularly acute for samples that are labelled with protons only on the exchangeable backbone amide positions, as the amide protons within an alpha helix are typically too far away from amide protons in other secondary structure elements to produce a detectable NOE. Consequently, labelling of only the amide protons of alpha-helical proteins leads to a restraint network that is too sparse to calculate a 3D structure. Other site-specific labelling schemes can supplement amide labelling, leading to a denser restraint network (Goto and Kay, 2000). Site-specific labelling of the terminal methyl groups of isoleucine, leucine, and valine (ILV-labeling) is particularly common (Tugarinov et al., 2006), but several alternatives and complementary labeling methods exist, e.g., Kainosho and Güntert (2009), Otten et al. (2010), and Gifford et al. (2011). While these additional labelling schemes can increase the density of the restraint network, they often come at the cost of increased complexity and the need to synthesize or purchase expensive precursors that are required to generate the isotope-labelled samples.

Paramagnetic NMR methods have emerged as potentially viable alternatives, capable of providing valuable information about electron-nucleus distances up to ∼20–30 Å (Koehler and Meiler, 2011; Pilla et al., 2017). Paramagnetic relaxation enhancement (PRE) experiments have been performed with native metalloproteins and proteins modified with covalent paramagnetic tags such as nitroxide spin labels and metal chelates (Bertini et al., 2005; Clore and Iwahara, 2009; Keizers and Ubbink, 2011). These techniques can be used to extend the scope of NMR methods to larger, more complex systems by providing long-range distances when short-range NOEs are unavailable or limited. Due to the long-range nature of paramagnetic relaxation enhancement, PRE experiments can provide valuable distance restraints even in sparsely labelled perdeuterated protein samples.

The utility of a distance restraint generally depends on two factors (Sullivan et al., 2003; Sullivan and Kuntz, 2004). First, restraints with short spatial distances are more valuable than those with long spatial distances because there are many more ways for two particles to be far apart than close together. Thus, a short-distance restraint provides more information than a long one. Second, this effect is more substantial for restraints involving residues that are more distant in the sequence. Thus, the most valuable restraints involve residues distant in sequence but close together in space. NOESY experiments provide powerful short spatial distance restraints (<6Å) but can miss many crucial long sequence distance restraints due to distribution of the isotope labels. In contrast, PRE experiments will yield more distance restraints due to the paramagnetic relaxation enhancement effect’s long-range nature. Many of these restraints will be of limited utility due to their long spatial distances; however, the collective effect of all of these long spatial distance restraints with the remaining short spatial distance (<12 Å) restraints can still be potent. As an aside, PRE methods have also become a popular approach to explore lowly populated transient protein states (Iwahara and Clore, 2006).

Using PRE data for 3D protein structure determination presents several challenges. First, each experiment only provides information about the spatial proximity of a given proton to a single site labelled with a paramagnetic tag. Adequately determining the 3D structure requires multiple experiments with different tag locations, increasing both experimental time and cost. Second, each experiment provides only a limited amount of information (Battiste and Wagner, 2000; Gottstein et al., 2012). Although a single experiment provides information about the spatial proximity of each residue to the paramagnetic tag, much of this information is redundant. For example, if a residue is close to the tag, then neighbouring residues in the sequence are also likely to be close. Furthermore, information that a residue is close to the tag provides a far more powerful structural constraint than information that a residue is distant from the tag, but the latter occurrence is far more frequent. Third, the derived distances can be imprecise due to intermolecular interactions, secondary metal-binding sites, and diamagnetic contamination (Clore and Iwahara, 2009). Fourth, heterogeneity and dynamics (Clore et al., 1990; Ryabov and Fushman, 2007; Bertini et al., 2012) can complicate the interpretation of PRE data. Relaxation can be strongly affected by conformational heterogeneity due to the inverse sixth power relationship between the PRE and the electron-nucleus distance; i.e., a minor structural population with a strong paramagnetic effect can have a significant impact on the measured data (Clore and Iwahara, 2009). Finally, the effects of spin diffusion due to dipole-dipole coupling can limit the accuracy of the measured PRE data (Vlasie et al., 2007; Vlasie et al., 2008; Bellomo et al., 2021). These challenges have slowed the widespread adoption of PRE-based methods for structure determination in favour of traditional NOE-based approaches.

Residual dipolar coupling (RDC) measurements are a common supplemental data source to PRE and NOE-based experiments. RDC measurements are carried out on systems where the protein is weakly aligned relative to the external magnetic field. Rather than reporting on distances, RDCs report on the angles between bonded atoms (typically backbone N-H bond vectors) and the external magnetic field, which provides valuable orientational information that complements distance information from PRE or NOE experiments (Prestegard et al., 2004). RDCs have been used for structure refinement and as restraints in *de novo* structure prediction software (Banci et al., 1998; Raman et al., 2010; Prestegard et al., 2014). While many protein structures based on RDC measurements have been reported, molecular modeling and low temperature annealing procedures are often used to derive and refine the 3D structures (Chou et al., 2000; Lipsitz and Tjandra, 2004; Huang and Vogel, 2012). Clearly there is room for more unbiased approaches to incorporate such RDC data into protein structure calculations.

Integrative approaches to structure determination (Ward et al., 2013) have emerged as practical tools for converting NMR and other experimental data into useful structural models. For example, PRE and RDC measurements have been used to drive molecular docking studies (van Dijk et al., 2005; Gelis et al., 2007; Gochin et al., 2011), as restraints in molecular dynamics simulations to generate ensembles of conformers (Dedmon et al., 2005; Asciutto et al., 2011), or they have been incorporated into Rosetta scoring functions (Lange et al., 2012; Kuenze et al., 2019). We recently demonstrated the structure determination of a small protein using PRE measurements in solid-state NMR (Perez et al., 2019). However, integrative methods are not without their own set of challenges. Even the most sophisticated methods can still struggle as the data becomes sparse, ambiguous, or unreliable, and considerable method development is often required to treat a new type of experimental data in order to correctly account for its characteristics, e.g., the conformational heterogeneity of spin-labels in PRE measurements (Iwahara et al., 2004; Anthis et al., 2011; Andrałojć et al., 2017).

Here, we show that these challenges can be overcome by using a sophisticated integrative structural biology approach called Modeling Employing Limited Data (MELD) (MacCallum et al., 2015). MELD combines experimental data from multiple sources with physical modelling to overcome the challenges of sparse, ambiguous, and difficult to interpret experimental data to infer accurate protein structural ensembles. We combine PRE and RDC measurements with secondary structure predictions based on backbone chemical shifts. We use MELD to infer the structure of Calmodulin in complex with the 20-residue smooth muscle myosin light chain kinase peptide (169 residues total). Calmodulin was selected for this exploratory work as it has an almost completely helical structure where the absence of inter-helical close contacts between amide protons makes 3D structure determination by NOE-based approaches difficult. Calmodulin-peptide complex have previously been used as models for integrative approaches using sparse NMR data (Andrałojć et al., 2014; Carlon et al., 2019). We show that MELD can identify conformations within 3 Å of a reference X-ray crystal structure using only sparse paramagnetic NMR restraints and RDCs from amide protons in combination with backbone chemical shifts, while successfully addressing conformational heterogeneity and noise in the NMR data.

## Experimental Methods

### Calmodulin–Peptide Complex as a Model System

In this work, we illustrate our approach for the protein calmodulin in complex with the smooth muscle Myosin Light Chain Kinase (smMLCK) peptide. Throughout, we will use a previously solved crystal structure of this complex [PDB ID: 1cdl (Meador et al., 1992)] as a reference.

### Overview of Labeling Strategy

**FIGURE 1:**
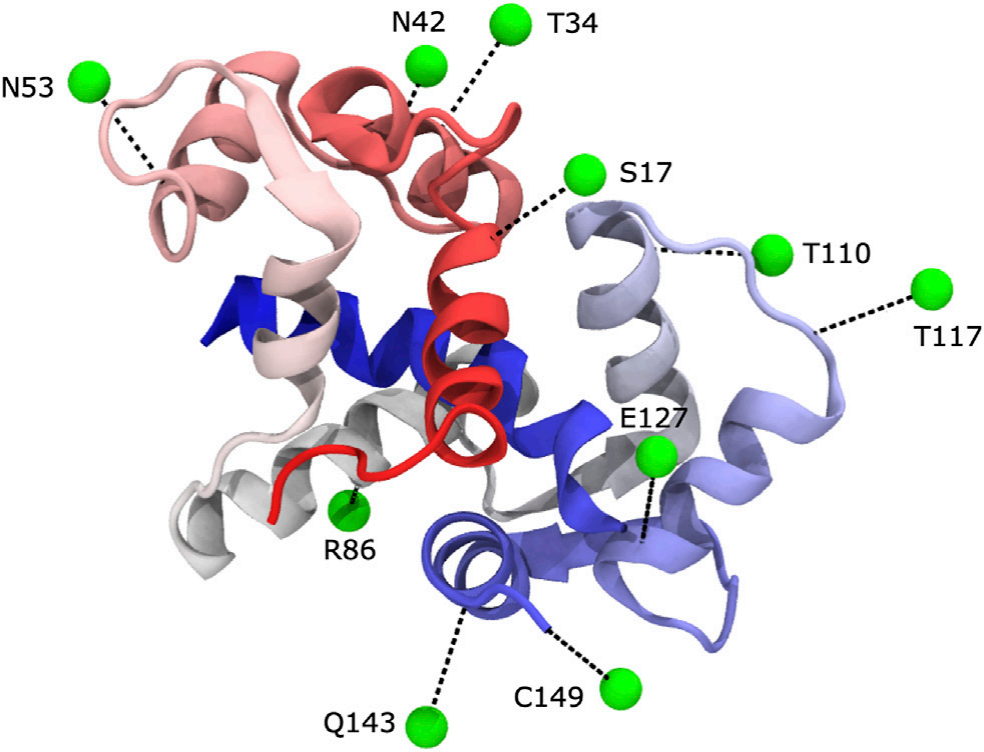
We carried out PRE experiments with ten different label sites. In each experiment, Calmodulin was MTSL-labeled at a different position. Spin labels were generally located in predicted surface-exposed sites within secondary structures. Spin labels are shown in green as virtual sites (see text), and their corresponding cysteine mutation linkage site is shown in black (PDB 1cdl).

In previous studies, specific nitroxide spin-labeled target peptides that bind to calmodulin were used; in this manner it was possible to map out the orientation of the peptide with respect to the protein (Zhang et al., 1995; Yuan et al., 2004). In this work, we collected PRE data for a total of ten spin-labelled protein sites (Figure 1). Nine of the sites on the protein were chosen to be solvent-exposed and within secondary structure elements by manual inspection of predicted secondary structure and solvent exposure. The remaining site, C149, was a single-residue extension of the C-terminus. To better simulate the process for a system without a previously determined structure, the protein’s known structure **was not used** in choosing the spin-labelled sites. Indeed, we learned later that several of the selected sites provided little useful information because they are either distant from the rest of the protein or they provided information that is mostly redundant with that obtained from other labeling sites.

To simulate the limited availability of isotopically labelled peptide, either due to cost or difficulty of production, only four of the ten spin-label data sets (chosen randomly) were collected with isotopically labelled peptide. The remaining six data sets were collected without labelled peptide, which results in the peptide being present but unlabeled and undetectable in the ^1^H, ^15^N HSQC NMR experiments.

### Protein Production

The ten single-cysteine point calmodulin (CaM) mutants (S17C, T34C, N42C, N53C, R86C, T110C, T117C, E127C, Q143C, 149C) were made by standard site-directed mutagenesis methods for attachment of the thiol-specific nitroxide spin label (1-oxyl-2,2,5,5-tetramethyl-δ-3-pyrroline-3-methyl) methanethiosulfonate (MTSL, Toronto Research Chemicals). Correctness of the mutations was confirmed by DNA sequencing. Calmodulin contains no Cys residues, so highly site-specific labeling can be obtained in this manner. MTSL is a relatively compact, yet highly reactive molecule compared to other commercially available nitroxide spin-labels that have been used to modify Cys residues; its shorter more rigid structure would be an advantage for the PRE studies [for discussion see for example (Guo et al., 2008; Fawzi et al., 2011)]. ^13^C and ^15^N-labeled CaM was expressed in M9 minimal medium with 99.9% ^15^NH_4_Cl and ^13^C_6_-glucose (0.5 gr/L and 3 gr/L, respectively; Cambridge Isotopes Laboratories) as isotope sources. Proteins were expressed and purified as described previously (Liu and Vogel, 2012; Ishida et al., 2016).

We followed a standard protocol for attaching the nitroxide spin-label to each single-cysteine CaM mutants with the spin-labelling reagent MTSL (Battiste and Wagner, 2000). To prepare the CaM/smMLCK complex sample, a 1.2-fold excess of either unlabeled or labelled peptide was mixed with each CaM mutant protein. All preparations were divided into two NMR samples. One sample was reduced to inactivate the spin-label by adding a 3-fold excess of ascorbic acid.

### Peptide Production

A construct with a 6xHis-KSI (D38A) fusion-protein tag was generated for smMLCK peptide expression in *Escherichia coli* (Jaroniec et al., 2005; Ishida and Vogel, 2010). The ketosteroid isomerase (KSI) coding sequence generates an insoluble protein, and this directs the protein-peptide fusion directly into inclusion bodies, where they are protected from proteolytic cleavage (Hwang et al., 2014). A linkage sequence “GGGGSSDP” with the Asp-Pro acid cleavage site was designed between the KSI protein and the sequence of the smMLCK peptide. The entire 6xHis-KSI-GGGGSSDP-smMLCKp gene sequence was inserted between the NdeI and XhoI sites of the pET15b(+) plasmid (Novagen), which was subsequently transferred into BL21(DE3) *E. coli* cells for protein expression. The cells were grown in either LB media (for unlabeled peptide) or minimal M9 media (containing ^13^C_6_-glucose and ^15^NH_4_Cl isotope to produce isotope-labeled peptide) and they were induced at OD_600_ = 0.6 with 1 mM IPTG for 4 h at 37°C. A cell lysate was prepared as previously described. The insoluble fusion protein was separated after one hour of centrifugation (18,000 rpm) and then resuspended in 6 M guanidine hydrochloride. Impurities were removed before the insoluble proteins can be extracted with metal chelate chromatography on a nickel affinity column. After extensive dialysis with double distilled H_2_O, the precipitated insoluble protein was collected and the Asp-Pro bond was cleaved in 10% formic acid at 80°C for 90 min (Hwang et al., 2014). The protein-peptide mixture was flash frozen with liquid nitrogen and lyophilized. Insoluble proteins and other impurities were removed after the lyophilized mixture was resuspended in a 20 mM Tris-HCl buffer (pH = 8.0). Finally, the unlabeled and isotope-labeled smMLCK peptides were purified with reverse-phase HPLC (COSMOSIL 5C_18_-AR-300, Nacalai United States). All purified peptides were lyophilized and stored at −20°C for further use. The final peptide sequence after cleaving is PARRKWQKTGHAVRAIGRLSS. The N-terminal proline is not observable in the NMR experiments and was not included in modeling with MELD.

### Chemical Shift Assignments

All NMR experiments were carried out on a 600 MHz Bruker AVANCE spectrometer with a field strength of 14.1 T. Backbone resonance assignments for the protein and the bound peptide were confirmed with the following 3D experiments: HNCO, HNCA, HNCOCA, HNCACB, and CBCA(CO)NH, as described previously (Liu and Vogel, 2012). All data were processed using NMRPipe (Delaglio et al., 1995) and analyzed with the program NMRView (Johnson and Blevins, 1994). All chemical shifts from these experiments were used to obtain backbone torsion angles from the program TALOS+ (Cornilescu et al., 1998; Shen et al., 2009). Secondary structure elements as identified through the assigned chemical shifts were as expected based on the known structure.

### Paramagnetic Relaxation Enhancement Measurements

Two ^1^H, ^15^N HSQC spectra were obtained for each spin-label construct. Each system contained each ^15^N-labeled protein and either unlabeled or ^15^N-labeled peptide, depending on the spin-label site (S17C, N53C, T127C, and 149C had isotopically labeled peptide). One HSQC was collected with active spin-label, whereas the other HSQC was collected with reduced, inactivated spin-label. The distances between the spin-label and the affected nuclei were calculated using the two-time point method (Iwahara et al., 2007).

### Residual Dipolar Coupling Measurements

Finally, to supplement the PRE experiments, we obtained RDC measurements for the amide groups in the complex with a sample where both the protein and peptide are isotopically labelled. Residual dipolar couplings (RDC) were measured for the CaM/smMLCK complex sample in a partially aligned media, which contains 2 mM bis-Tris (pH = 7.0), 300 mM KCl and 16 mg/ml Pf1 bacteriophage (Asla Biotech Ltd.). The IPAP-HSQC experiment was used for the RDC measurements (Ottiger et al., 1998). In these experiments the effects of dipole-dipole cross-correlated relaxation can impact the accuracy of ^1^J_NH_ splitting measured from the spectra introducing a small residual bias in the RDCs. While these systematic errors can be eliminated by using a selectively-decoupled sequence (Yao et al., 2009), the errors are small relative to the magnitude of the measured RDCs and are expected to have a minimal effect on structure determination (Yao et al., 2009). Our work uses only a single RDC alignment. Notably, a mutant of Calmodulin is capable of selective binding to lanthanides, which provides a strategy for the measurement of multiple RDCs (Bertini et al., 2009). A quantitative assessment of protein mobility/heterogeneity by RDC would require the use of multiple alignments (Barbieri et al., 2002; Tolman, 2002; Bouvignies et al., 2006; Higman et al., 2011; Guerry et al., 2013; Andrałojć et al., 2015). However, as discussed further below, it is not currently possible to conduct such an analysis with the MELD approach, as MELD compares individual structures, rather than ensembles of structures, to the experimental data.

## Computational Approach

### Overview of Modeling Employing Limited Data Approach

Here, we employ MELD, a physics-based Bayesian approach for structural determination to infer the ensemble of structures most consistent with the known physics of protein structure and experimental data (MacCallum et al., 2015; Perez et al., 2015). MELD uses a Bayesian framework to combine a physics-based prior distribution with a data likelihood function to make statistically consistent inferences about conformations that explain the experimental data.

MELD uses Bayes’ theorem:

p(x|D)∝p(x)p(D|x), (1)

where x represents the atomic coordinates and D represents the data. The physics-based prior, p(x), specifies which structures are more likely *a priori* and determines the distribution of structures in the absence of data. In the present study the physics-based prior is given by the Amber ff14SB force field (Maier et al., 2015) with a grid-based torsion potential (Perez et al., 2015) and the OBC generalized-Born implicit solvent model (Onufriev et al., 2004). The likelihood function, p(D|x), captures the compatibility between the data and some structure x. In MELD, the likelihood function takes the form of a unique restraint function (MacCallum et al., 2015), explained in more detail below. The goal of Bayesian inference is to compute the posterior distribution, p(x|D), which is the most statistically consistent inference given the prior, likelihood, and data.

As discussed in the Results section below, the term *ensemble* is highly overloaded in structural biology and care is required in interpretation. MELD belongs to the class of methods where a single structure, rather than entire ensemble of structures, is considered in the likelihood function, such that each member of the ensemble individually agrees with the experimental data. Any conformational heterogeneity (e.g., flexible loops) may represent true intrinsic heterogeneity, but may also simply reflect a lack of data. As such, MELD produces a form of *uncertainty ensemble* in the terminology of Bonomi et al., 2017.

### Overview of Experimental Data

The input to our approach is: 1) the protein sequence, 2) TALOS+ secondary structure predictions derived from backbone chemical shifts (Cornilescu et al., 1998; Shen et al., 2009; MacCallum et al., 2015), 3) distance restraints derived from PRE measurements, and 4) orientational restraints derived from RDC measurements. We have recently demonstrated the success of a similar approach for PRE measurements in solid-state NMR (Perez et al., 2019).

**FIGURE 2:**
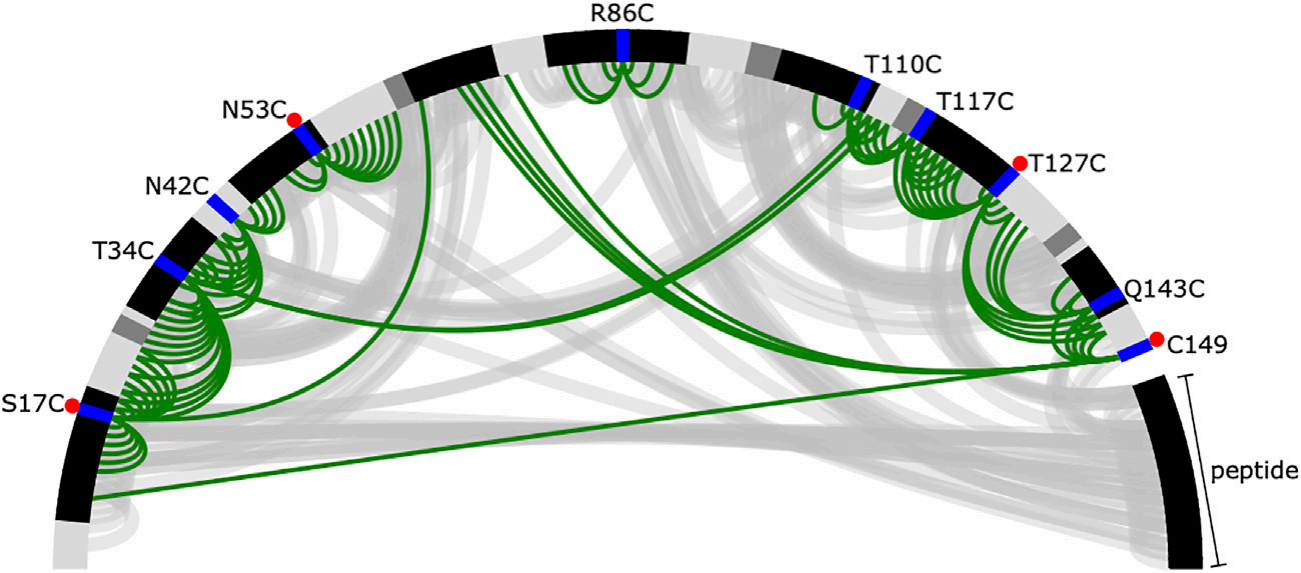
Summary of short distances from the reference crystal structure and inferred from the PRE data. The protein and peptide backbones are represented in a half-circle where the colour depends on the secondary structural element (black—helix, dark grey—extended, light grey—loop). The spin-label locations are shown in blue. A red dot indicates the experiment was performed with labelled peptide. Short distances [ (*I, j)* pairs where |i−j|>4 and $r_{ij}^{C\alpha }<7.6\;$Å] derived from the crystal structure are shown as grey arcs. Short distances ($r_{ij}^{label-NH}<12\;$Å) derived from the PRE data are shown in green. Note that C149 is a single-residue extension of calmodulin, which is 148 residues long.

PRE data is often both noisy and sparse (Kim et al., 2014), which makes structural inference challenging. Despite collecting data for ten spin-label positions, we can derive only a few distance restraints that are short in spatial distance (in this case, we define short as <12 Å) (Figure 2). Of these short spatial distances, only a small number correspond to residues that are distant in sequence, which would provide the most information about folding (Gottstein et al., 2012). Furthermore, as stated previously, to simulate the limited availability of isotopically labelled peptide, only four of ten datasets (S17C, N53C, T127C, and 149C) had labelled peptide, and there are no short distance PREs between the peptide and the protein. This leaves the peptide’s correct placement to be dictated by longer, less informative spatial distance restraints and the physical model, which makes accurate inference more challenging.

### Deriving Distances From Paramagnetic Relaxation Enhancement Data

Our first step was to develop a consistent method to convert ensemble-averaged PRE measurements into distance restraints. PRE data were turned into approximate distances using the Solomon-Bloembergen equations following the standard approach (Battiste and Wagner, 2000; Iwahara et al., 2007).

For nitroxides, Curie-spin relaxation is negligible and the transverse relaxation enhancement, Γ_2_, is dominated by direct dipole-dipole interactions (Clore and Iwahara, 2009). In this case, ${\text{Γ}}_{2}$ is related to the distance between the paramagnetic center and the observed nucleus, *r*:

$$\Gamma_{2}=\frac{K}{r^{6}}\left[4\tau_{c}+\frac{3\tau_{c}}{1+\;\omega_{H}^{2}\tau_{c}^{2}}\right]$$

where ω_H_ is the Larmor frequency of the proton, and τ_c_ is the correlation time for the electron-nuclear interaction defined as $\tau_{c}=\;\;{\left(\tau_{r}^{-1}\;+\;\tau_{s}^{-1}\right)}^{-1}$ where τ_r_ is the rotational correlation time, and τ_s_ is the electron relaxation time. Previous experiments (Lee et al., 2002) have determined that $\tau_{c}\approx 9.5\;ns$ for Calmodulin in complex with smMLCK peptide at 25°C. For nitroxides, the electron relaxation time is long (Iwahara et al., 2007) ($\tau_{s}>10^{-7}s$), so the rotational correlation time dominates and $\tau_{c}\approx \tau_{r}\approx 9.5\;ns$. *K* is given by:

$$K=\frac{1}{15}\;{\left(\frac{\mu_{0}}{4\pi }\right)}^{2}\gamma_{1}^{2}g^{2}\mu_{B}^{2}S\left(S+1\right)$$

where µ_0_ is the permeability of vacuum, γ_1_ is the nuclear gyromagnetic ratio, *g* is the electronic g factor, µ_B_ is the Bohr magneton, and *S* is the electron spin quantum number. Γ_2_ can be estimated using a two-point time measurement (Iwahara et al., 2007):

$${\text{Γ}}_{2}=R_{2,para}-R_{2,dia}=\frac{1}{\text{Δ}T}\ln\frac{I_{dia}\left(T_{b}\right)I_{para}\left(T_{a}\right)}{I_{dia}\left(T_{a}\right)I_{para}\left(T_{b}\right)}$$

where ΔT is a time delay chosen to minimize the error in Γ_2_, and I_dia_ and I_para_ are the peak intensities for the diamagnetic and paramagnetic samples, respectively (Iwahara et al., 2007). In this work, we use ΔT=20 ms.

### Incorporating Paramagnetic Relaxation Enhancement Information Into Modeling Employing Limited Data Calculations

The distances derived from PRE data correspond to ensemble averages with an *r* ^−6^ weighting, but in MELD (and most other structure determination software), restraints are applied to single structures rather than ensembles. To account for conformational heterogeneity of both the protein and the flexible spin-label, the PRE-derived distances are turned into flat bottomed harmonic restraints that allow for a range of distances without penalty. This approach is a tradeoff that ensures that individual structures are not erroneously over-restrained but this can allow discrepancies between the measured and modelled ensemble averages. Our aim is to produce an approximately correct ensemble starting from an extended chain. If desired, the resulting ensemble can be further refined using a variety of ensemble approaches (Boomsma et al., 2014; Hummer and Köfinger, 2015; Bonomi et al., 2016; Gaalswyk et al., 2018).

We divided the data into *short*, *medium*, and *long* distances with corresponding upper and lower bounds (Table 1). *Short* and *long* distances are difficult to quantify with precision because the peak is either completely broadened for residues close to the spin-label or barely changes intensity for those that are far away. These distances are turned into broad restraints that either start from zero or extend to infinity for *short* and *long*, respectively. *Medium* distances correspond to peak intensity changes that can be quantified more precisely and are turned into restraints centered around the predicted value. All distances include a buffer of ±5 Å of the measured distance to account for the flexibility of the spin-label and noise in the experimental data (Perez et al., 2019).

**TABLE 1:** Distance bounds for calculated PRE distances, *r*.

	PRE distance range (Å)	Restraint upper bound (Å)	Restraint lower bound (Å)
Short	*r* ≤ 12	17	0.0
Medium	12 < *r* < 20	*r* + 5	*r* − 5
Long	*r* ≥ 20	∞	15

Ranges are chosen based on the nature of the PRE and include a +/− 5 Å buffer to account for heterogeneity and flexibility.

Due to noise in the experimental data, partially overlapping peaks, and instantaneous fluctuations in both the protein structure and the position of the spin label, we observed that restraints are sometimes violated even with a ±5 Å buffer. To mitigate this issue, we used MELD’s unique ability to require that only a certain fraction of the restraints must be satisfied by each structure. We set this *active fraction* to 0.9. Essentially, as long as 90 percent of the restraints are satisfied, the resulting restraint energy will be zero. We treat the remaining restraints as being derived from spurious data, so they are entirely ignored. Every timestep, MELD decides which restraints are active based on the current structure. Further details can be found in the SI and in MacCallum et al. (2015).

In our approach, the various hyperparameters (boundaries between *short*/*medium*/*long*, size of buffer, active fraction) are fixed. One potential improvement would be to place a hyperprior on these values and infer them using an extended Bayesian approach like Inferential Structure Determination (Rieping et al., 2005). This would allow the data and physical model to determine the most likely values of these hyperparameters, rather than requiring their specification *a priori*. As MELD does not currently support inference of hyperparameters, we chose the simpler approach of setting a wide buffer and lower active fraction, which potentially sacrifices a small amount of information.

The spin-label was modeled using virtual sites (Banci et al., 1996) following the approach of Islam and Roux (Islam et al., 2013; Islam and Roux, 2015). These virtual sites represent the spin-label as a non-interacting dummy particle to simplify the simulation without losing relevant information for structural refinement. These simplified dummy nitroxide spin-labels are parameterized to match the spin-labels’ 3D spatial distribution and dynamics in all-atom simulations. The virtual sites are non-interacting, allowing us to account for all ten spin labels in a single simulation without the risk of interactions between them.

Secondary structure restraints were derived from TALOS+ (Cornilescu et al., 1998; Shen et al., 2009) and used to restraint MELD simulations as previously described (MacCallum et al., 2015). Our approach works by first breaking the protein into overlapping 5-residue fragments. If 4/5 of the residues in the fragment are predicted to be helical or extended, then the fragment is restrained using a combination of torsion and distance restraints (MacCallum et al., 2015). All secondary structure restraints are then combined into a collection with an active fraction of 0.95, which allows 5 percent of fragments to differ from their predicted secondary structure.

### Incorporation of Residual Dipolar Coupling Information Into Modeling Employing Limited Data

The traditional approach to incorporate RDCs into simulations is based on solving for the optimal alignment tensor, which requires solving a system of equations every time step using singular value decomposition (SVD) or related methods, which can be computationally intensive (Losonczi et al., 1999). We found this to be particularly problematic in the GPU-accelerated framework of MELD, where this traditional approach led to a 300 percent increase in run time (data not shown), primarily due to the extreme speed of the rest of the force/energy calculations and the challenge of efficiently parallelizing SVDs for small systems of equations on a GPU. To mitigate this issue, we instead followed the approach in Habeck, Nilges, and Riepling (Habeck et al., 2008), which we implemented using an OpenMM CustomCentroidBondForce (Eastman et al., 2017). In our implementation, the alignment tensor elements are encoded in two non-interacting dummy particles coupled to the rest of the system through an additional energy term. This approach has two benefits. First, it is dramatically faster than the standard approach on GPUs, with negligible cost compared to the calculation of the non-bonded forces. Second, this approach accounts for uncertainty and produces a joint distribution of alignment tensors and structures, providing a Bayesian posterior estimate of the conformational ensemble that better reflects uncertainty. A full explanation of our implementation can be found in the SI. To account for uncertainty in the experimental data and to avoid erroneously over-restraining individual structures to the ensemble average data, we use a flat-bottomed restraint where the energy is zero if the computed RDC is within 1.5 Hz of the measured value. Another approach that avoids the need to solve for the alignment tensor is given in Camilloni and Vendruscolo (2015).

## Results and Discussion

### Interpretation of Modeling Employing Limited Data-Computed Ensembles

The term “ensemble” is highly overloaded in structural biology, with a variety of meanings in different contexts (Bonomi et al., 2017; Andralojc and Ravera, 2018; Gaalswyk et al., 2018). Care must be taken to ensure correct interpretation.

MELD samples from a well-defined conformational ensemble (Gaalswyk et al., 2018), specifically the Bayesian posterior distribution given by Eq. 1. Interpretation of this ensemble is straightforward: it is the statistically consistent posterior distribution inferred from the prior, likelihood, and data. How should one select or report structures from this ensemble? The approach we take here is simply to report all structures, as this fully captures the heterogeneity of the distribution. If there is a limit to the number of structures reported, one simple, correct approach is to select a subset of structures at random. Alternatively, one could cluster the structures and report the cluster medoids and populations along with some measure of the variance of structures within the cluster. A variety of approaches are supported by the PDB-Dev archival system which is being developed for structural models obtained using integrative modeling (Vallat et al., 2018). However, we note that since MELD samples structures with the correct posterior probabilities, it is incorrect to further select structures based on other criteria, such as selecting the lowest energy structures.

A second consideration in ensemble interpretation is the nature of the likelihood function. The experimental measurements are averages over a thermodynamic ensemble. The most correct modeling approach is to use a likelihood function that considers an entire ensemble of models, ensuring that the predicted average quantities match their corresponding experimental measurements (Bonomi et al., 2017; Andralojc and Ravera, 2018; Gaalswyk et al., 2018). This is an ill-defined inverse problem (Bonomi et al., 2017; Andralojc and Ravera, 2018), so regularization is required, typically in the form of physical modelling and entropy maximization (Bonomi et al., 2017; Andralojc and Ravera, 2018; Gaalswyk et al., 2018). While conceptually appealing, ensemble likelihood methods are complex with high computational requirements. Alternatively, most methods in structural biology, including the MELD approach described here, use single-structure likelihoods (Boomsma et al., 2014). These methods are overly restrictive, as they require each member of the ensemble to be consistent with the data to within some tolerance. In the current approach, we use relatively wide tolerances, but this still does not guarantee that that the computed ensemble accurately models the true distribution.

The primary issue is that for a given set of experimental measurements, there are many possible ensembles that could produce it. The ensemble that MELD generates ensures that each structure is in reasonable agreement with the data and allows for a reasonable degree of flexibility. However, the MELD average might not precisely match the experimental measurement due to the use of wide tolerances. Furthermore, the true ensemble could be “broader” than the one generated by MELD—the true ensemble could have many structures that are individually in poor agreement with the data, while still having the same ensemble average, see Figure 1 of Gaalswyk et al. (2018) for a simple illustration. In the terminology of Bonomi et al. (2017), MELD produces an *uncertainty ensemble*, where heterogeneity in the calculated ensemble could represent true heterogeneity in the system or could simply reflect a lack of data for some part of the protein. The single-structure approach is likely reasonable when the true ensemble has only a modest amount of heterogeneity, e.g., small fluctuations around an average structure, but could be expected to break down for highly heterogenous systems, e.g., systems containing intrinsically disordered regions.

Although we do not pursue it here, a promising approach would be to use a method like MELD to compute an initial approximate ensemble that could then be used as a starting point for ensemble approaches (Boomsma et al., 2014; Hummer and Köfinger, 2015; Bonomi et al., 2016; Gaalswyk et al., 2018).

### The Accuracy of Inference Depends on the Protocol Used

To determine how the experimental data should be incorporated, we performed several simulations varying in their set up (Trial1–Trial4). We explored various ways of combining the restraints into *collections* (Table 2). In MELD, at every timestep, the restraints in a collection are sorted by energy, and the *active fraction* with the lowest energy are “active” and contribute their forces and energy to the system, while the remainder are “inactive” and ignored. The division of restraints into collections matters because it determines how MELD decides which restraints are active and which are ignored. For example, Trial1 combines all of the restraints into a single collection. In this case with an active fraction of 0.9, MELD can freely ignore any 10 percent of the restraints, which could be, for example, ignoring one of the ten spin labels entirely. Trial2 separates the restraints by spin-label and into *short*, *medium*, and *long-distance* ranges, resulting in 30 collections. Now MELD can only ignore 10 percent from each spin label/distance combination, while the remaining 90% will be active. Trial3 extends Trial2 by adding the RDC restraints. Trial1–Trail3 start from an extended conformation generated by the tleap tool from the AmberTools suite (Case et al., 2021). Trial4 follows the same protocol as Trial3 but starts from the reference crystal structure as a control.

**TABLE 2:** Grouping of restraints for simulations and description of individual trials.

Trial	Number of collections	Description
Trial 1	1	All PRE restraints in a single collection
Trial 2	3 × 10 = 30	PRE restraints are combined by spin-label position and distance (Short, Medium Long) into 30 collections.
Trial 3	3 × 10 = 30	PRE restraints are combined by spin-label position and distance (short, medium long) into 30 collections.
		RDC restraints are included.
Trial 4	3 × 10 = 30	PRE restraints are combined by spin-label position and distance (short, medium long) into 30 collections.
		RDC restraints are included.
		Simulation starts from native crystal structure.

PRE restraints have a force constant of 250 kJ mol^−1^ nm^−2^. RDC restraints have a force constant of 0.5 kJ mol^−1^ Hz^−2^.

For each trial, we ran a 2.5 µs replica exchange simulation using 48 replicas. The temperature and the force constant for each restraint collection varied across replicas (see SI for details). The last 0.5 µs of the lowest replica was used for analysis.

### Using Only Paramagnetic Relaxation Enhancement-Derived Information Leads to Modest Structural Quality

**FIGURE 3:**
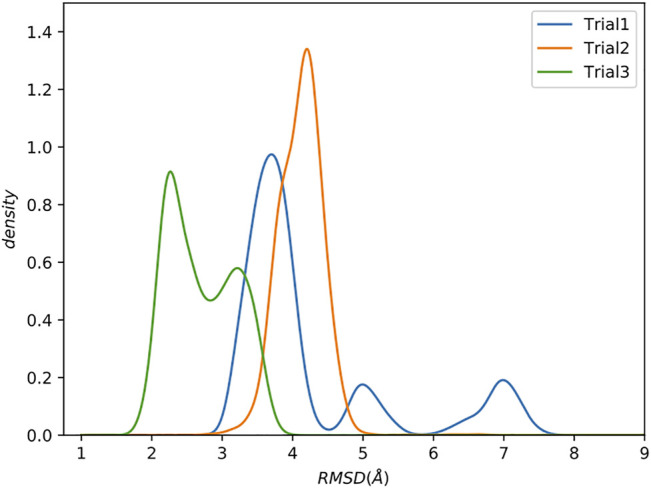
Increasing complexity of how restraints are incorporated results in better sampling. Kernel Density Estimate (KDE) plot of backbone RMSD to the reference structure. The last 0.5 µs were analyzed. Flexible tails at the N- and C-termini of the protein were excluded.

We compare the trials using kernel density estimation plots (KDE; see Supporting Information for details) of the backbone root mean square deviation (RMSD) to the reference structure [PDB: 1cdl (Meador et al., 1992)], excluding the flexible tails at the N and C terminals of the protein which are not present in the reference (Figure 3).

Trial1 is the most straightforward approach and combines all of the data into a single collection. Many of the structures have relatively low RMSD to the reference (<4Å), which is promising considering the rather limited experimental data, but there are also structures with much higher RMSDs of ∼5 Å and ∼7.5 Å RMSD. There are various explanations for these high RMSD conformations, but perhaps the simplest is that this way of grouping all restraints into a single collection allows MELD to ignore short spatial distance restraints that would otherwise eliminate these conformations. As previously stated, the utility of a restraint depends on its spatial distance. Shorter distances provide highly constraining information. However, this highly constraining nature means that these restraints are more difficult to form, leading MELD ignore them in favour of more easily satisfied restraints.

To test this hypothesis, in Trial2, we further subdivided the restraints by separating the *short*, *medium*, and *long* restraints from each dataset into separate collections, resulting in 30 total collections.

The resulting RMSD distribution is centered at a modest RMSD of ∼4 Å, which is slightly worse than the mode from Trial1. However, this method of combining restraints into collections has wholly eliminated the high RMSD conformations.

Although the RMSDs obtained are only modest (∼4 Å), these results were obtained with a very sparse dataset with only one spin-label per 17 amino acids. This equates to 6.4 total restraints per residue, and only 0.8 short-distance restraints per residue. For context, NOE-based structures from fully protonated samples typically have >15 NOE restraints per residue, all with short distances.

Based on visual examination, several of our spin-label sites appear to give restraints that are largely uninformative, either because they are far from the remainder of the protein (e.g., R86C) or because they are mostly redundant with other spin-label positions (e.g., T110C), see Figures 1, 2. Our results could be improved with a more judicious choice of the 10 label sites, but it is unclear how to do this without pre-existing knowledge of the structure. The results could likely be improved further by adding additional spin-label sites using the calculated structural ensemble to optimize probe location, although we do not pursue this here. Such an iterative strategy could be a viable approach to improve model accuracy but comes at an additional experimental cost. More rigid spin-labels (Fawzi et al., 2011) could also improve results, as MTSL still displays significant conformational heterogeneity that results in less precise distance restraints.

### Residual Dipolar Couplings Provide Complementary Information That Improves Accuracy

Despite collecting data for 10 different spin-label sites, few yielded informative short spatial distance, high sequence distance restraints (Figure 2), limiting the models’ achievable accuracy to relatively modest RMSDs of around 4 Å. Rather than collect additional PRE data, we instead chose to explore the utility of combining PRE information with residual dipolar couplings (RDCs) measured for the amide groups.

**FIGURE 4:**
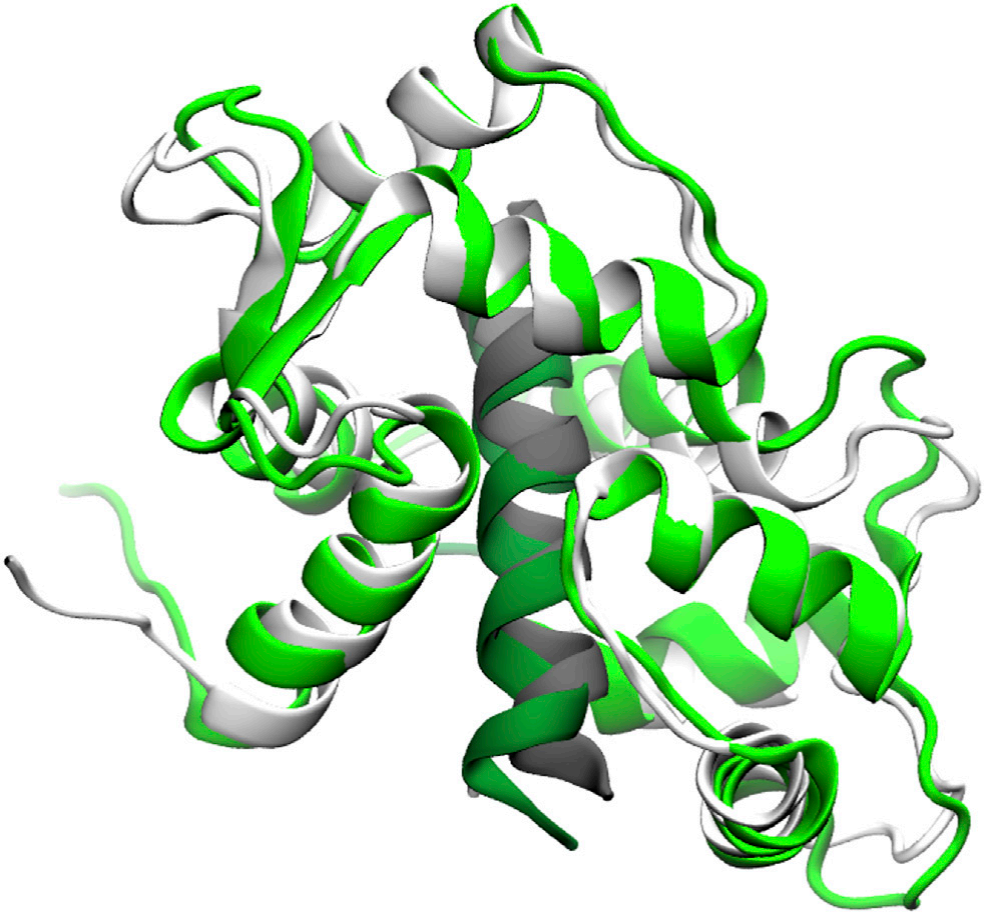
Superposition of a typical model (green) from the Trial3 ensemble with the reference structure (white). Peptide is shown in dark green and grey respectively. The superposition was over residues 4–146 of calmodulin plus the peptide. The backbone RMSD of this structure is 2.8 Å, which is near the mean of the ensemble. Structures with RMSDs as low as 1.6 Å are sampled.

Residual dipolar couplings provide information about the orientation of amide NH bonds complementary to the distance information from PRE experiments. In Trial3, we combined PRE information (using the same strategy as Trial2) with RDC data. The inclusion of RDC data led to a substantial improvement in the RMSD (Figure 3). The RMSD ranges from approximately 1.6–4.0 Å with an average RMSD of 2.8 Å, including both Calmodulin and the smMLCK peptide (Figure 4). This improvement of RMSD upon inclusion of RDC data is consistent with previous studies showing that RDC data provides valuable information on the relative orientation of the two lobes of calmodulin (Mal et al., 2002; Gifford et al., 2011).

RDCs provide information about how the amides are oriented, which, when combined with secondary structure restraints (derived from the measured backbone chemical shifts) and distance restraints (derived from PREs), serves to dramatically limit the possible structures that simultaneously agree with the experimental data and the physical model.

To assess the potential quality of sidechain packing, we examined the single best structure obtained during our simulations, which has an RMSD of 1.6 Å (Supplementary Figure S1). For this “best” structure, the RMSD for sidechain heavy atoms is 2.1 Å for all sidechains and 1.4 Å for core sidechains. This is notable as there are no restraints on the side chains themselves, only between the spin labels and the backbone amide protons. This packing phenomenon with MELD has been noted previously and can be attributed to the accuracy of the physical model (Perez et al., 2019). However, we note that the sidechain and backbone RMSDs are generally correlated, and this structure has a lower backbone RMSD than average, so the average side chain RMSDs will be higher than these figures.

### Despite Limited Data, the Peptide Is Routinely Placed Correctly

As noted previously, the experimental data contained no short distance PREs to the peptide, so placement of the peptide is dependent on a combination of *medium* and *long* restraints with the physical model. Furthermore, only 4 of 10 experiments contained labelled peptide, with the peptide undetected in the remaining experiments. Nevertheless, the combination of available data and the physical model was still able to routinely position the peptide correctly (the peptide is included in the RMSD calculations shown in Figure 3). The structure of calmodulin depends on the peptide and its binding (Barbato et al., 1992; Ikura et al., 1992), so correct placement of the peptide is critical.

### The Individual Lobes Are Better Defined Than the Complex

**FIGURE 5:**
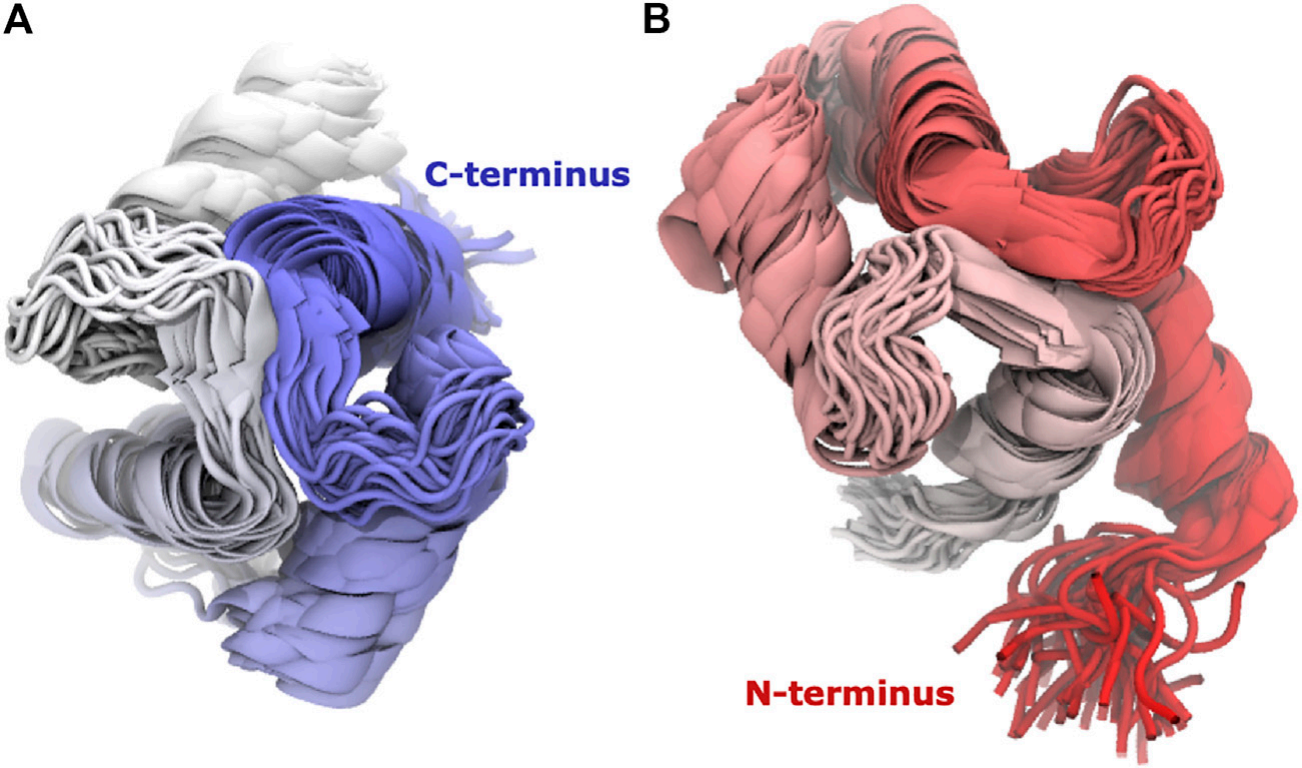
The domains have tightly clustered ensembles. Superpositions of **(A)** C-lobe (residues 82–149) and **(B)** N-lobe (residues 1–76) of Calmodulin for Trial3. Every 100th frame from the last 0.5 microseconds is shown, coloured from N-terminus (red) to C-terminus (blue).

**FIGURE 6:**
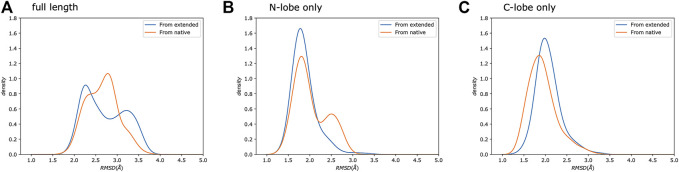
Simulations from extended and native show similar distributions. We show a comparison of the same protocol started from either an extended chain (Trial3, blue) or from the reference crystal structure (Trial4, orange). Each panel shows the backbone RMSD compared to the reference crystal structure. We compare: **(A)** the full-length protein, as well as the **(B)** N-, and **(C)** C-lobes.

Calmodulin consists of two lobes connected by a flexible linker that becomes structured upon peptide binding (Barbato et al., 1992). Examination of each lobe individually shows that our modeled ensembles are tightly clustered (Figure 5), indicating that most of the heterogeneity in our calculated ensemble arises from the relative motion of the two lobes. If we consider the RMSD of each lobe to the reference individually, the results are consistently lower than for the whole protein (Figure 6). The RMSD of the C-lobe to the reference structure is ∼2.1 Å (Figure 6B), which is consistent with typical RMSDs for small globular proteins seen in MD simulations. The results for the N-lobe are similar. The resulting heterogeneity in the relative orientation of the two domains should be interpreted with caution, due to the use of a single-structure likelihood, as discussed above.

A previous study (Carlon et al., 2019) examining the joint X-ray/NMR refinement of Calmodulin in complex with the Death-Associated Protein kinase (DAPk) peptide revealed poor agreement between X-ray and NMR data due to large interprotein contacts in the crystal that stabilize a conformation that is in poor agreement with the solution NMR data. The crystal structure of the full-length DAPk protein in complex with Calmodulin lacks these contacts and is in much better agreement with the NMR data. These results highlight the need for caution when comparing structures determined by X-ray crystallography and NMR, particularly in cases where flexibility can be expected.

A study of 109 pairs of NMR and crystal structures (Sikic et al., 2010) showed that typical C⍺ RMSDs range from ∼0.5 to 4 Å with a mean of ∼2.0 Å when using the DALI (Holm and Sander, 1993) alignment. The typical variability of models within a given NMR ensemble was similar (Sikic et al., 2010). Our results for the individual lobes of calmodulin give similar average RMSDs, indicating that our approach is producing results comparable to typical NMR structures using NOEs and fully protonated samples despite the substantial sparsity in our data. Our results for the full-length complex produce a slightly higher average RMSD, which reflects heterogeneity in the exact relative placement of the two lobes.

To further test our predictions’ quality, we also ran calculations using the same protocol as Trial3 but starting from the reference crystal structure rather than from an extended chain (Figure 6), which sets a bound on the possible accuracy that could be obtained. The resulting RMSD distributions are similar to our predictions. This indicates that given: 1) the available experimental data, 2) potential limitations of the physical model used, 3) the use of a single-structure rather than ensemble likelihood, and 4) the challenges of comparing with a static crystal structure, the results obtained using MELD are essentially as good as they could be.

### Computational Requirements

Each calculation was over 48 replicas for 2.5 µs, which required approximately 6 days on 48 GTX 1080Ti GPUs. However, examination of the RMSD over time (Supplementary Figure S2) shows that the simulations appear to be converged after ∼500 ns. In hindsight, the simulation length could have been reduced to 1 µs without a loss in quality, which would reduce simulation time to 2.5 days. While computationally expensive, our approach is readily feasible with access to advanced research computing or cloud computing resources.

## Conclusion

Our approach can generate accurate protein structures starting from an extended chain using backbone chemical shifts in combination with PRE and RDC measurements from backbone amide labeled samples. We demonstrate this on a relatively large, complex system with only one spin label per 17 residues. This gives an average of 6.4 PRE restraints per residue of which less than 0.8 per residue are short-distance, compared to the >15 short-distance restraints per residue that are typical in NOE-based structure determination. Our approach is able to routinely identify dominant conformations within 3 Å of the reference crystal structure for calmodulin in complex with a peptide and correctly places the peptide despite a lack of information relating the peptide to the protein. These results approach the quality of gold-standard, fully protonated NMR structures based on NOEs, but were obtained from a far sparser dataset using methods that are more applicable to large proteins. The inclusion of RDCs highlights their value in structure determination with minimal PRE-derived distance restraints. These results showcase the importance of spin label location and the effect it has on the value of the resulting restraints. We show that MELD can accurately account for challenges related to conformational heterogeneity and noise and achieve moderate side chain packing. These results also highlight the capabilities of integrative approaches when experimental information is limited.

## Data Availability

Restraint files, run scripts, and analysis scripts can be found on our github repository (https://github.com/maccallumlab/ calmodulin_pre_paper) and are archived with Zenodo (DOI: 10.5281/zenodo.5071079).
