# Supplementary material for: An Integrative Approach to Determine 3D Protein Structures Using Sparse Paramagnetic NMR Data and Physical Modeling
Source: Front Mol Biosci. 2021 Aug 12;8:676268. doi: 10.3389/fmolb.2021.676268 (PMC8407082; doi:10.3389/fmolb.2021.676268)
Supplement: Supplementary file 1 [file DataSheet1.PDF]

1 Structure Determination

1.1 Setup of MELD Simulations

MELD[1] was used to generate the structural models from NMR data. MELD is based upon OpenMM[2], an open-source GPU-accelerated simulation engine, All simulations were 2.5 microseconds. Proteins were modeled using the ff14SB[3] force field plus AMAP[4] torsion potential correction with the GB^{OBC} implicit solvent model[4]. A combined Hamiltonian and temperature replica exchange approach was used[1] with 48 replicas. The force constants for PRE restraints were "turned on" from zero to 250 kJ mol^{-1} nm^{-2} following the schedule in Table 1. The force constants remained at full strength for the remaining replicas. The force constant for RDC restraints were "turned on" from zero to 0.5 kJ mol $^{-1}$ Hz^{-2} following the schedule in Table 1. The temperature was geometrically decreased from 550 K to 300 K following the schedule in Table 1. Temperature was kept constant at 550 K for the remaining replicas. Restraint strength was ramped from 0 to full for the first 100/200 steps, following the schedule in Table 1. Replica exchanges were attempted every 50 ps.

Trial	PRE scaler (α)	RDC Scaler (α)	Temp scaler (α)	Ramp (steps)	Description
1	0.5-1	N/A	0-0.5	1-100	All
2	S 0.5-1<br>M 0.4-0.5<br>L 0.3-0.4	N/A	0-0.5	1-100	By position and by distance (Short, Medium, Long)
3	S 0.6-1<br>M 0.5-0.6<br>L 0.4-0.5	0.3-0.4	0-0.3	1-200	By position and by distance (Short, Medium, Long).<br>With RDCs
From extended	S 0.6-1<br>M 0.5-0.6<br>L 0.4-0.5	0.3-0.4	0-0.3	1-200	By position and by distance (Short, Medium, Long).<br>With RDCs<br>From extended.

Table 1: Schedule for replica exchange ladder. The force constants for PRE and RDC restraints were turned on the upper part of the ladder while temperature was geometrically scaled across the bottom part of the ladder.

In MELD we utilize a 1-dimensional exchange ladder where we vary temperature and the force constants of the restraints. We define α [0,1] which varies across the ladder; the lowest replica has α =0, the highest α =1. We start with evenly spacing the α values across the ladder. For every parameter that can vary (i.e. temperature and force constant), we can express them as a function of α, T(α), k(α). The value of α determines the value of those parameters.

Scripts for running the MELD simulations (including experimental data) can be found at the Github repository `https://github.com/maccallumlab/calmodulin_pre_paper`.

1.2 Implementation of RDC Restraints

In traditional NMR approaches, calculation of dipolar couplings requires knowledge of degrees of alignment and of average orientation of the molecule[5]. But in *de novo* structure determination the alignment tensor is *a priori* unknown. Therefore estimates of axial and rhombic components of alignment

tensor are used instead. Axiality and rhombicity are characteristics of a symmetric 2nd rank tensor in 3D Euclidean space describing its orientational asymmetry[6] and can be represented in a matrix.

The traditional approach to estimate the elements of the alignment tensor is to use anisotropic spin interactions by determining the Saupe order matrix via singular value decomposition (SVD) to factorize a real complex matrix thereby solving the Ax+b equation[7]. A Saupe matrix is symmetric and traceless leading to only five independent elements.

The approach of Habeck, Nilges, and Rieping[8] involves applying Bayes theorem to derive a joint posterior probability distribution for atomic coordinates, tensor elements, and errors of the data set. The probability distribution is determined by observed dipolar couplings and a few basic assumptions. This probability distribution quantifies the interdependence of different groups of parameters and tells us how they can be used to estimate parameters from the data. Finding and exploring regions of high posterior probability through statistical sampling will result in high precision estimates. Their approach starts with modeling the observation of a single dipolar coupling with a Gaussian error distribution. The likelihood function quantifies how consistent the settings for features such as error or conformational degrees of freedom are with the parameters. The prior comes from a canonical ensemble at $1/T\beta$ and is based on the standard molecular force field. The posterior is used to generate a sequence of sequential samples, from which the estimates are taken.

Our approach is based on that of Habeck, Nilges, Rieping[8]. Rather than solving for the exact alignment tensor at every step (like the SVD approach), we sample from a distribution of alignment tensors. The five components of the alignment tensor are encoded in the positions of two dummy atoms relative to the center of mass. There is a restraint on the z-component of the second dummy particle to ensure that it does not diffuse off into infinity.

Hyperparameters include the quadratic cut (s^{-1}) which is the deviation beyond which restraints become linear, and the scale factor which scales the value of kappa and the alignment tensor to the coordinates of the dummy particles. This will result in the alignment tensor being scaled up by the scale factor and kappa being scaled down. Ideally the largest values of the scaled alignment tensor are on the order of 1e-4, therefore the default value is 1e-4. The value of scale factor must be the same for all experiments that share an alignment.

The dummy particles have a mass of 12.0, charge of 0.0, type of SDUM, are named S1 and S2, with a solvent radius of 1.0, and screen of 0.5. Observed dipolar couplings are in Hz. A tolerance is included such that the calculated couplings within the tolerance (Hz) of the observed dipolar couplings will have zero energy and force. The force constant will be in kJ/mol/Hz^2. Typical kappa values are:

1H-1H: -360300 Hz A^3
13C-13C: -90600 Hz A^3
15N-1H: 36500 Hz A^3

The system consists of the atoms of the system plus two dummy particles. The atoms interact through the usual atomic potential. The coordinates of the dummy particles are coupled to the bond vectors involved in RDCs using the expressions derived in Habeck, Nilges, Rieping[8]. This energy term was implemented as an OpenMM `CustomForce`[2], which enables automatic parallelism on GPUs. Sampling of the combined system using a standard stochastic dynamics integrator results in drawing samples from the joint distribution over alignment tensors and molecular structures.

1.3 Definition of Calmodulin Lobes

The lobes of calmodulin are defined as: N-lobe (residues 1-76), C-lobe (residues 82-147), flexible linker (77-81)[9]. The flexible tails are residues 1-4 and 148-149 which are not present in the crystal structure[10] and were eliminated from analysis. In simulations the peptide is defined as residues 153-172.

2 Analysis

All computational analyses were performed excluding the tail regions of the protein and including the peptide and used the last 0.5 μs of simulation. Relevant scripts can be found on the Github repository.

2.1 KDEs

KDE plots were generated for the backbone RMSD to native structure using scikit-learn[11] with a bandwidth of 0.1 and a Gaussian kernel to control the kernel shape and smoothness of the distribution.

2.2 Side-Chain Packing

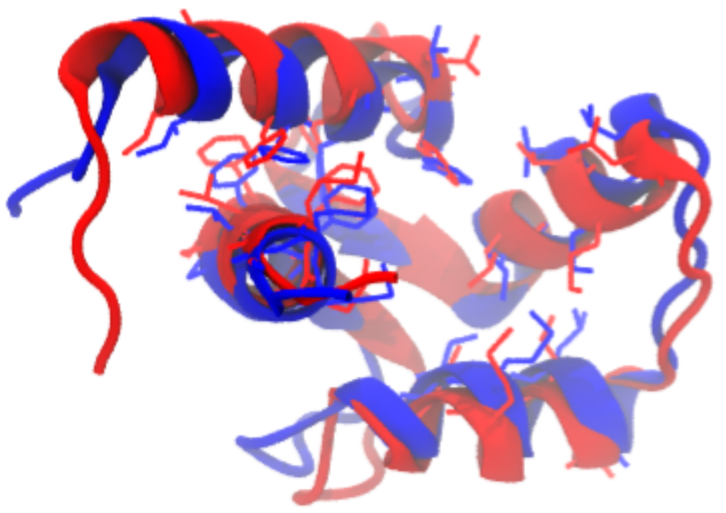

Figure S1: Side chain packing of core side chains in N lobe from best structure in Trial 4.

2.3 Convergence

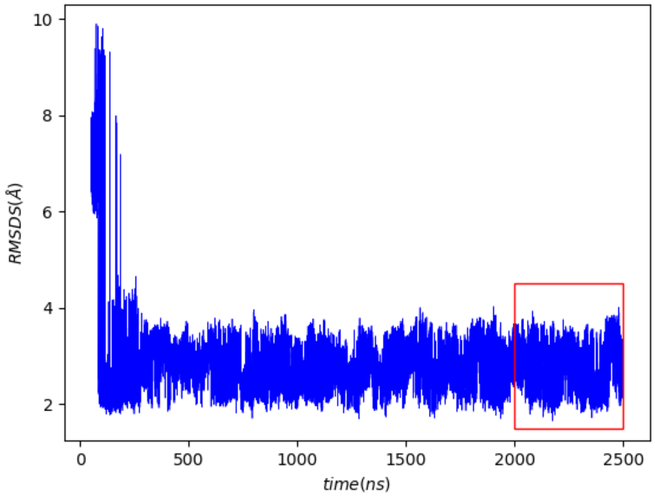

Figure S2: Backbone RMSD between Trial3 simulation and the reference structure over residues 4-146 of Calmodulin and all residues of the peptide. The red box indicates the section of the trajectory used for analysis (last 0.5 µs).

Supplemental References

(1) MacCallum, J. L.; Perez, A.; Dill, K. A. Determining Protein Structures by Combining Semireliable Data with Atomistic Physical Models by Bayesian Inference. *Proc. Natl. Acad. Sci.* **2015**, *112* (22), 6985–6990. https://doi.org/10.1073/pnas.1506788112.

(2) Eastman, P.; Swails, J.; Chodera, J. D.; McGibbon, R. T.; Zhao, Y.; Beauchamp, K. A.; Wang, L.-P.; Simmonett, A. C.; Harrigan, M. P.; Stern, C. D.; Wiewiora, R. P.; Brooks, B. R.; Pande, V. S. OpenMM 7: Rapid Development of High Performance Algorithms for Molecular Dynamics. *PLOS Comput. Biol.* **2017**, *13* (7), e1005659. https://doi.org/10.1371/journal.pcbi.1005659.

(3) Maier, J. A.; Martinez, C.; Kasavajhala, K.; Wickstrom, L.; Hauser, K. E.; Simmerling, C. Ff14SB: Improving the Accuracy of Protein Side Chain and Backbone Parameters from Ff99SB. *J. Chem. Theory Comput.* **2015**, *11* (8), 3696–3713. https://doi.org/10.1021/acs.jctc.5b00255.

(4) Onufriev, A.; Bashford, D.; Case, D. A. Exploring Protein Native States and Large-Scale Conformational Changes with a Modified Generalized Born Model. *Proteins Struct. Funct. Bioinforma.* **2004**, *55* (2), 383–394. https://doi.org/10.1002/prot.20033.

(5) Prestegard, J. H.; Bougault, C. M.; Kishore, A. I. Residual Dipolar Couplings in Structure Determination of Biomolecules. *Chem. Rev.* **2004**, *104* (8), 3519–3540. https://doi.org/10.1021/cr030419i.

(6) Chen, K.; Tjandra, N. The Use of Residual Dipolar Coupling in Studying Proteins by NMR. *Top. Curr. Chem.* **2012**, *326*, 47–67. https://doi.org/10.1007/128_2011_215.

(7) Losonczi, J. A.; Andrec, M.; Fischer, M. W. F.; Prestegard, J. H. Order Matrix Analysis of Residual Dipolar Couplings Using Singular Value Decomposition. *J. Magn. Reson.* **1999**, *138* (2), 334–342. https://doi.org/10.1006/jmre.1999.1754.

(8) Habeck, M.; Nilges, M.; Rieping, W. A Unifying Probabilistic Framework for Analyzing Residual Dipolar Couplings. *J. Biomol. NMR* **2008**, *40* (2), 135–144. https://doi.org/10.1007/s10858-007-9215-1.

(9) Anthis, N. J.; Doucleff, M.; Clore, G. M. Transient, Sparsely Populated Compact States of Apo and Calcium-Loaded Calmodulin Probed by Paramagnetic Relaxation Enhancement: Interplay of Conformational Selection and Induced Fit. *J. Am. Chem. Soc.* **2011**, *133* (46), 18966–18974. https://doi.org/10.1021/ja2082813.

(10) Meador, W. E.; Means, A. R.; Quiocho, F. A. Target Enzyme Recognition by Calmodulin: 2.4 A Structure of a Calmodulin-Peptide Complex. *Science* **1992**, *257* (5074), 1251–1255. https://doi.org/10.1126/science.1519061.
(11) Pedregosa, F.; Varoquaux, G.; Gramfort, A.; Michel, V.; Thirion, B.; Grisel, O.; Blondel, M.; Prettenhofer, P.; Weiss, R.; Dubourg, V.; Vanderplas, J.; Passos, A.; Cournapeau, D. Scikit-Learn: Machine Learning in Python. *J. Mach. Learn. Res.* **2011**, *12*, 2825–2830.
